# Atomic-Scale In Situ Scanning Transmission Electron Microscopy of MoS_2_ during Lithiation

**DOI:** 10.1021/acsnano.5c05218

**Published:** 2025-07-21

**Authors:** Kei Nakayama, Shunsuke Kobayashi

**Affiliations:** Nanostructures Research Laboratory, 92043 Japan Fine Ceramics Center, Nagoya, Aichi 456-8587, Japan

**Keywords:** scanning transmission electron microscopy, in situ, lithiation, MoS_2_, structural dynamics

## Abstract

Atomic-scale in situ characterization techniques are necessary to clarify the dynamic local structural changes during intercalation reactions. High-resolution transmission electron microscopy (HRTEM) is at the forefront of this field. However, the image contrast in HRTEM is not always straightforward, necessitating the exploration of alternative imaging techniques. Here, using annular dark-field (ADF) scanning transmission electron microscopy (STEM), which is expected to provide a more directly interpretable image contrast, we report atomic-scale in situ observation of the MoS_2_ lithiation process induced by electron irradiation. Using a single-crystalline specimen, dedicated specimen holder, and alternate low- and high-magnification image acquisition technique, a time series of ADF-STEM images was recorded at a frame rate of 1 fps, followed by image filtering. Consequently, successive changes in the domain structure were clarified, starting with an anisotropically expanding structural phase transition, followed by the disappearance and formation of domains with different crystal orientations, which were attributed to changes in the internal stress and interfacial energy.

## Graphical abstract

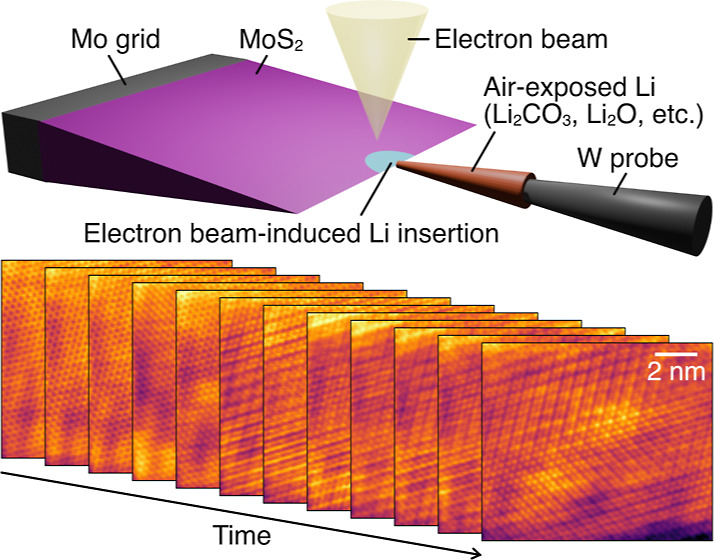

In situ characterization of materials provides a unique opportunity to directly observe dynamic changes within materials, which is challenging to achieve with ex situ methods. Among various characterization techniques, transmission electron microscopy (TEM) and scanning transmission electron microscopy (STEM) are particularly advantageous due to their high spatial resolution, which extends from the nanoscale to the atomic scale.^1,2^ Leveraging this strength, TEM and STEM have revealed real-space dynamic microstructural evolutions associated with intercalation reactions of atoms such as Li,^3–16^ which are crucial in applications such as catalysts,^17^ capacitors,^18^ and batteries.^19–21^ However, despite the atomic-scale imaging capabilities of TEM and STEM, in situ observation of intercalation reactions at this scale remains less developed compared with lower-magnification studies, limiting a deeper understanding of atomic-scale dynamic phenomena during these reactions.

For atomic-scale in situ observation of intercalation reactions, a promising technique is in situ high-resolution TEM (HRTEM), which has been used to successfully visualize the movement of the reaction front during the lithiation process of MoS_2_.^12^ However, it is worth noting that the image contrast of HRTEM is not always straightforwardly interpretable. HRTEM gives an interference pattern with the same symmetry as that of the projected atomic arrangement. However, this pattern is not always sufficient to determine the projected atomic arrangement. Furthermore, the HRTEM image contrast can depend on the defocus value and sample thickness. Therefore, it is often necessary to acquire a defocus series of experimental images and compare them with a defocus-thickness series of simulated images. However, during in situ observations, it is not possible to acquire a defocus series of images for a given structure because the structure itself changes when images are taken at different defocus values. This problem necessitates the development of alternative techniques. For example, annular dark-field (ADF) STEM provides more directly interpretable incoherent *Z*-contrast images (where *Z* is the atomic number), owing to its image formation mechanism, which employs scattered electrons with phase correlations averaged over a wide range of scattering angles.^2,22^ However, to the best of our knowledge, atomic-scale in situ STEM for lithiation processes has not yet been achieved, which hinders the advancement of electron microscopy studies on dynamic lithiation processes.

Here, using ADF-STEM, we report an atomic-scale in situ observation of the MoS_2_ lithiation process induced by electron irradiation. MoS_2_ was chosen because it is a prototypical layered transition metal dichalcogenide that can accommodate Li.^23^ Upon lithiation, it undergoes a structural phase transition involving a change in layer stacking sequence, volume expansion, and symmetry lowering—features that are of fundamental interest in understanding intercalation-induced structural dynamics. Furthermore, MoS_2_ has been widely studied as a candidate electrode material for batteries,^24,25^ making its lithiation behavior highly relevant to energy applications. These characteristics make MoS_2_ an ideal model system for probing dynamic intercalation processes at the atomic scale using in situ ADF-STEM. Although several TEM studies on MoS_2_ lithiation have been reported,^6,7,12,14,15^ time-series imaging at the atomic scale has been demonstrated only in a single study using HRTEM.^12^ Even in that case, however, it remains challenging to resolve atomic-scale structural dynamics due to the complexity of interpreting interference patterns and overlapping moiré contrast. In this study, with the aid of a single-crystalline specimen, dedicated specimen holder, and alternate low- and high-magnification image acquisition technique, a time series of atomic-scale ADF-STEM images was obtained with a frame rate of 1 fps, followed by image filtering. As a result, successive changes in the domain structure were clarified, starting with the anisotropically expanding structural phase transition,^23^ and followed by the disappearance and formation of domains with different crystal orientations, which are attributed to the changes in internal stress and interfacial energy.

## Results and Discussion

Figure 1a illustrates a schematic of the experimental setup. Air-exposed Li (the main composites were Li_2_CO_3_ and Li_2_O; see Figure S1) was brought into contact with single-crystalline MoS_2_ under a microscope. The electron beam was used not only to acquire images but also to induce Li insertion into MoS_2_. The electron beam is crucial in providing energy for the following possible reactions: MoS_2_ + 1/2 Li_2_CO_3_ → LiMoS_2_ + 1/2 CO_2_ + 1/4 O_2_ and MoS_2_ + 1/2 Li_2_O → LiMoS_2_ + 1/4 O_2_, which do not occur spontaneously. Further details on the experimental setup are provided in the Supporting Information (Note S1; Figures S2–S6; and Movies S1 and S2). Figure 1b–d shows images obtained using in situ ADF-STEM at low magnification (more images are provided in Movie S3). The contrast change progressed to the interior of the MoS_2_ specimen from the area where air-exposed Li came into contact. This change was attributed to the bending and cracking of the specimen originating from the volume expansion due to Li insertion. Electron energy-loss spectroscopy (EELS) analysis consistently revealed that Li was inserted into MoS_2_, as presented in Figure 1e,f.

**Figure 1:**
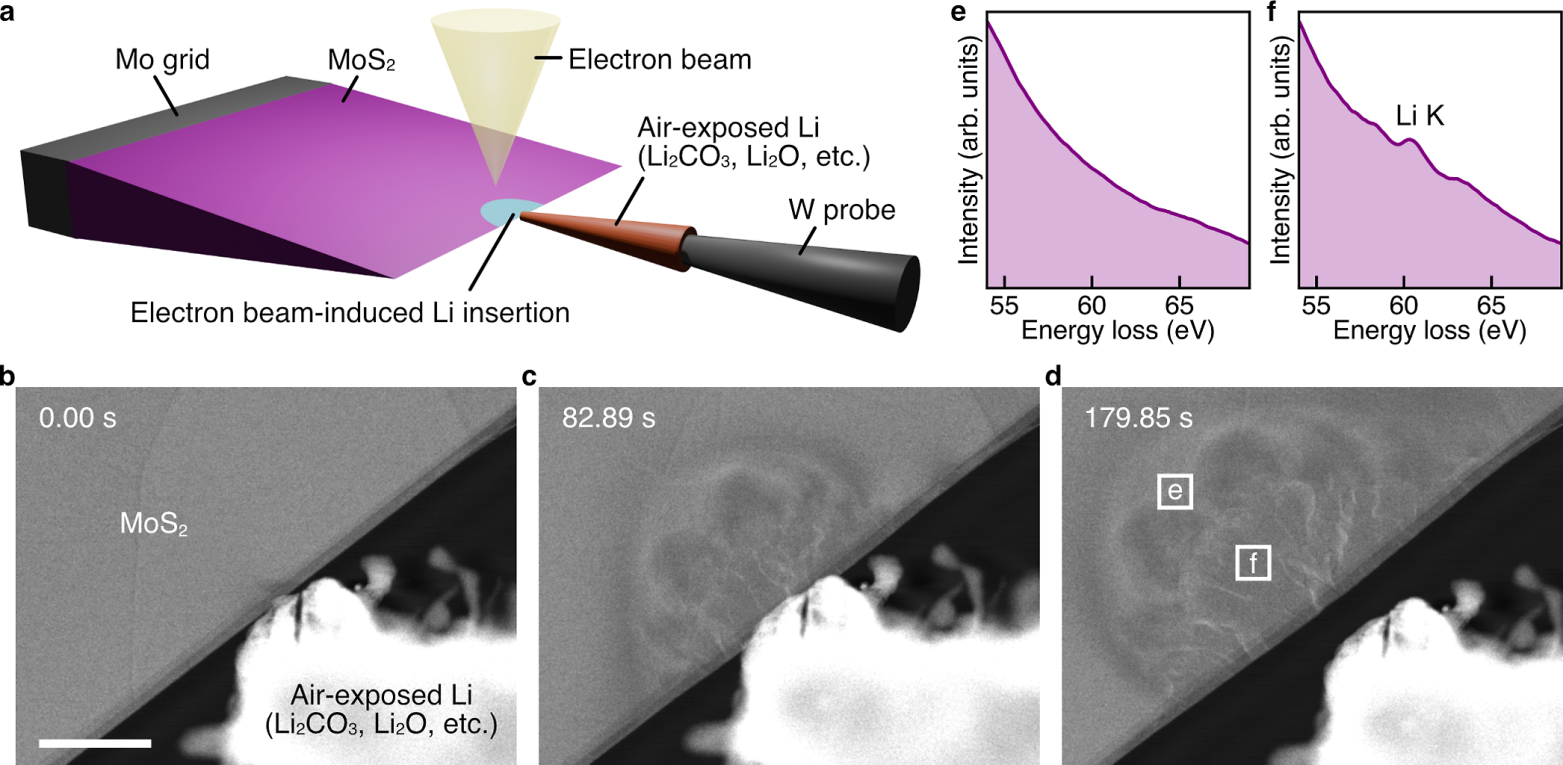
In situ ADF-STEM at low magnification. (a) Schematic of the experimental setup. (b–d) Snapshots at 0.00, 82.89, and 179.85 s (e,f) EEL spectra obtained from the square regions in (d). Scale bar, 500 nm (b).

When attempting to perform a similar observation at high magnification, a challenging situation arises: the electron beam must be irradiated on the air-exposed Li to induce Li insertion (see Figure S2 and Movie S1); nevertheless, the air-exposed Li vanishes quickly under high-magnification conditions because of the relatively high electron dose rate (a typical value is 4 × 10^6^ e^–^ nm^–2^ s^–1^). To avoid this, an alternate low- and high-magnification image acquisition technique was adopted (Figure 2a). Low-magnification images were acquired to moderately irradiate electrons (2.3 × 10^3^ e^–^ nm^–2^ s^–1^) around the interface between the MoS_2_ specimen and air-exposed Li, inducing Li insertion. High-magnification images (3.7 × 10^6^ e^–^ nm^–2^ s^–1^) were acquired to observe the structural changes occurring in MoS_2_, with electron irradiation limited to the MoS_2_ area. The acquired images are shown in Movie S4. Note that the low-magnification images are bright-field (BF) STEM images and not ADF-STEM images. The frame rate of the original data was approximately 2 fps, and thus, the frame rate for both the high- and low-magnification images was approximately 1 fps. Because the acquisition time (and electron dose) for each image was less than one-tenth that of conventional ex situ observations, the obtained high-magnification images were very noisy. Therefore, to reduce noise, threshold filtering in Fourier space, image alignment, and moving average filtering with a window size of two were applied (see Methods). The processed images are provided in Movie S5 and Figure S7, and some are presented in Figure 2b–h, where Figure 2d,e shows enlarged images of the rectangular regions in Figure 2c. The image contrast in Figure 2b (0.00 s) and the upper right of Figure 2c (16.74 s) corresponds to the 2H structure^26^ of MoS_2_, as indicated by the overlaid structural model in Figure 2d. The lower left corner of Figure 2c shows a different image contrast, which is attributed to the 1T″-Li_*x*_MoS_2_ structure^23^ as presented in Figure 2e, indicating that Li originated from outside the lower left corner of the field of view. The 1T″ structure may correspond to the structure denoted as 2 × 2 1T′ in ref 12 and 1T (2 × 2) in ref 14. Upon closer inspection of Figure 2e, a slightly thicker dark stripe contrast is observed, as marked by the white lines. As presented in the simulated image in Figure S8, this contrast corresponds to the (001) plane. Figure 2c shows two stripe-contrast orientations, as marked by the white lines. This indicates that two 1T″ regions with different crystal orientations were present, partially overlapping each other. As shown in Figure 2f (19.69 s), the area of the 1T″ phase increased, indicating the progress of lithiation. Only one stripe-contrast orientation was observed, as marked by the solid white lines, suggesting that the domain with the other crystal orientation disappeared. An out-of-phase boundary was also noted around the center of the image, as indicated by the dashed lines, likely serving as a mechanism to relieve the internal stress increased by volume expansion due to Li insertion. In Figure 2g (26.59 s), a new domain with a different crystal orientation appeared, as marked by the lines and dashed ellipse. Furthermore, in Figure 2h (43.34 s), an additional domain appears, as marked by the lines and dashed ellipse. Overall, the series of ADF-STEM images in Figure 2c,f–h indicates that during the MoS_2_ lithiation process, the domain structure successively changes once the 1T″ structure is formed.

**Figure 2:**
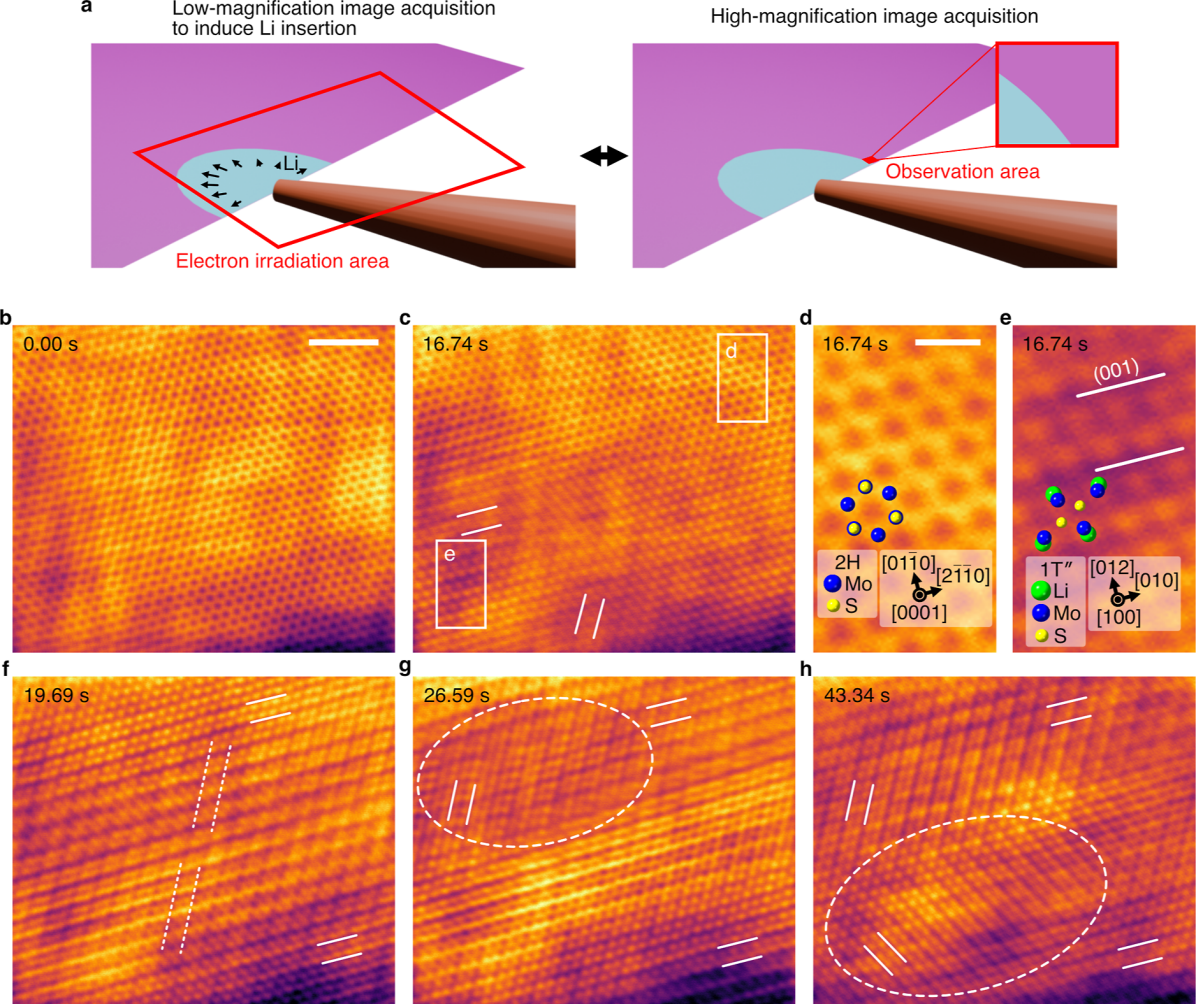
In situ ADF-STEM at high magnification. (a) Schematic of the image acquisition method. Low-magnification and high-magnification images were alternately acquired one by one. (b–h) High-magnification images processed by threshold filtering in reciprocal space, image alignment, and moving average filtering. Scale bars, 2 nm (b) and 0.5 nm (d).

Figure 3a,b illustrates schematics of the 2H and 1T″ structures. While the 2H structure has the hexagonal *P*6_3_/*mmc* symmetry,^26^ the 1T″ structure has a lower symmetry as a result of anisotropic lattice expansion due to Li insertion.^23^ For example, twice the spacing of the {0110} planes (0.548 nm) of the 2H structure corresponds to the three larger spacings of the crystallographically inequivalent (001) (0.588 nm), (010) (0.578 nm), and (011) (0.577 nm) planes of the 1T″ structure. The actual symmetry of the 1T″ structure is *P* 1, but here we approximate it as *P*2/*m* by assuming a 2-fold symmetry around the [100] direction (that is, treating β = 90.010° and γ = 90.077° as 90°). Because the space groups *P*6_3_/*mmc* and *P*2/*m* correspond to point groups 6/*mmm* and 2/*m*, respectively, the 2H to 1T″ transition may lead to six orientations of 1T″ domains. Three of them are illustrated in Figure 3b–d, referred to as Orientations I, II, and III, respectively, whereas the other three were obtained by rotating them 180° around the [010] direction. Orientation I is suggested to be present in the upper and lower right of Figure 2h, whereas Orientations II and III are suggested to be present in the upper and lower left, respectively, based on the arrangement of the bright spots and orientation of the dark stripe contrasts. However, it should be noted that the dark stripe contrasts with different orientations in Figure 2h overlap, indicating that the regions with Orientations I, II, and III overlap when viewed along the observation direction. A possible three-dimensional domain structure model for this situation is shown in Figure 3e,f and Movie S6, where the *z*-axis is parallel to the observation direction. The model thickness of 15.0 nm was adopted based on the specimen thickness estimated from EELS measurements (see Note S2). The simulated image in Figure 3g, calculated from the model in Figure 3e,f, reproduces the features shown in Figure 2h. Although this reproducibility alone cannot determine the three-dimensional domain structure, it strongly suggests that Figure 2h contains 1T″ regions with Orientations I, II, and III. Figure 3h illustrates the Mo and S arrangements in Orientation I overlaid on those in Orientation II. This schematic illustrates how the crystal orientation of the 1T″ structure can be altered through slight, diffusionless atomic displacements, as indicated by the arrows, functioning as ferroelastic switching.^27^ This suggests that local crystal orientations can easily change in response to changes in the internal stress during the MoS_2_ lithiation process. For example, the appearance of new domains, as shown in Figure 2f–h, can be understood through the schematics in Figure 3i–k. Figure 3i illustrates a situation in which a 1T″ domain is created, owing to lithiation. As the 2H to 1T″ transition causes volume expansion, the compressive stress increases, as indicated by the arrows. In particular, the compressive stress normal to the 1T″ (001) plane is likely larger than those along the other directions considering that the increase in the lattice spacings from the bulk 2H to bulk 1T″ structures takes the maximum for the 1T″ (001) plane (7.5%).^23,26^ This claim is additionally supported by an estimate of the lattice expansion based on experimental images (Figure S9). When the lithiation process progresses further, the 1T″ domain may grow (Figure 3j), but this growth further increases the compressive stress normal to the 1T″ (001) plane. Because the strain energy includes a term proportional to the sum of the squares of the internal-stress components,^28^ it can be effectively reduced by isotropically distributing the compressive stress through the formation of new domains with different crystal orientations (Figure 3k). Note that the creation of a new domain generates interfacial energy around it; therefore, the actual domain formation likely depends not only on the internal stress but also on the interfacial energy. The effects of the internal stress and interfacial energy are likely to compete with each other in the MoS_2_ lithiation process, because of the annihilation of a domain, which can be attributed to a reduction in the interfacial energy, as observed in Figure 2c,f.

**Figure 3:**
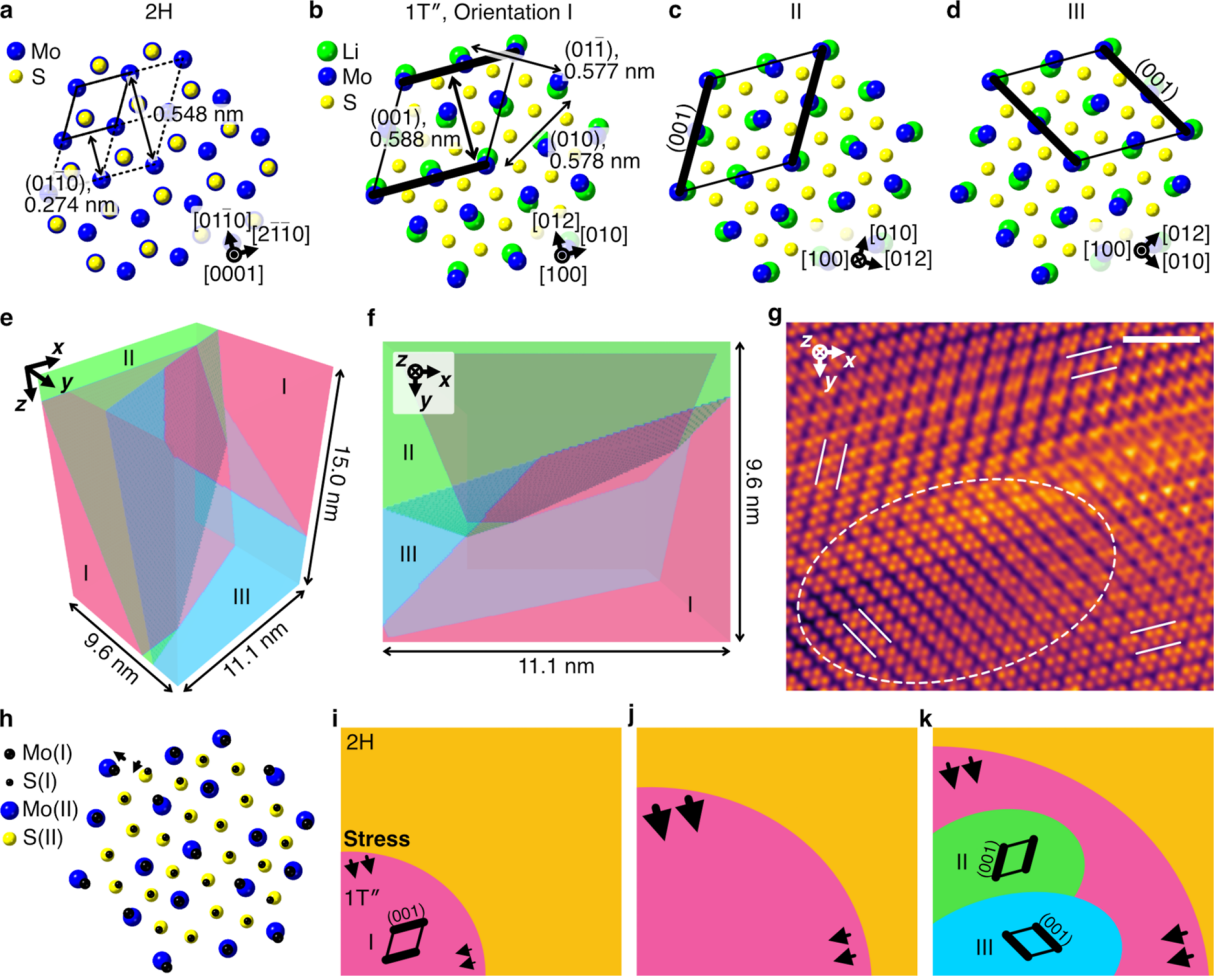
Discussion on the microstructural evolution during the MoS_2_ lithiation process. (a–d) Schematic views of the 2H and 1T″ structures. Solid and dashed lines indicate the unit cells, where the bold lines in (b–d) highlight the (001) plane of the 1T″ structure. (e,f) Schematics of a possible domain structure for Figure 2h in perspective views. The pink, green, and blue regions have the 1T″ structure with Orientations I, II, and III, respectively. (g) Simulated ADF-STEM image based on (e) and (f). (h) Mo and S arrangements of the 1T″ structure in Orientation I, overlaid on those in Orientation II. (i–k) Schematics for the appearance of new domains. Scale bar, 2 nm (g).

## Conclusions

In summary, we reported atomic-scale in situ ADF-STEM of the MoS_2_ lithiation process with the aid of a single-crystalline specimen, a dedicated specimen holder, and alternate low- and high-magnification image acquisition technique. Consequently, it was found that 1T″ domains with different crystal orientations can successively disappear and form during the process, after the 1T″ structure was initially formed. This flexible change in the domain structure is likely a key factor enabling MoS_2_ to accommodate large local volume changes and the resulting changes in internal stress due to lithiation. Further studies using atomic-scale in situ STEM may clarify the dynamic processes involved in intercalation reactions across a broader range of materials.

## Methods

### Electron Microscopy

An electron-transparent thin MoS_2_ flake was peeled off from a single crystal (ALLIANCE Biosystems, Inc.) with tweezers and attached to a Mo grid with an adhesive. The grid was placed on a specimen holder equipped with a piezo-driven W probe (Mel-Build Corporation). After attaching Li metal to the tip of the probe, the specimen holder was transferred to a microscope (JEM-ARM300F2, JEOL Ltd.) operating at 80 kV. During this process, the Li was exposed to air for approximately 30 min. The air-exposed Li was brought into contact with the MoS_2_ specimen under a microscope, where a voltage of 0 V was applied using an SP-200 potentiostat (Biologic). For ADF-STEM, the probe-forming aperture and detector semiangle were 24.5 mrad and 50–180 mrad, respectively. For in situ observation at low magnification (Figure 1), images were acquired at a frame rate of approximately 1.4 fps. The probe current was 220 pA, and the electron dose rate was 1.8 × 10^2^ e^–^ nm^–2^ s^–1^. For in situ observation at high magnification (Figure 2), low- and high-magnification images were alternatively acquired at a frame rate of about 2 fps using an in-house script for the microscope control, which was made with reference to ref 29. The probe current was 70 pA, and the electron dose rate was 2.3 × 10^3^ e^–^ nm^–2^ s^–1^ for low-magnification imaging and 3.7 × 10^6^ e^–^ nm^–2^ s^–1^ for high-magnification imaging. EEL spectra were obtained in the STEM mode using a GIF Continuum spectrometer (Gatan, Inc.), equipped with the microscope. For MoS_2_, 0.15 eV per channel was used, and the energy resolution was 0.6 eV (full-width at half-maximum of the zero-loss peak). For air-exposed Li, 0.4 eV per channel was used, and the energy resolution was 1.6 eV. The convergence and collection semiangles were 24.5 and 70 mrad, respectively. The background signals for the C K- and O K-edges in air-exposed Li were determined by using power-law fitting and subsequently subtracted.

### Image Processing

High-magnification images were Fourier transformed, processed with threshold filtering, and then inverse Fourier transformed. The threshold was set to 8.7 × 10^–4^ times the maximum intensity of all the Fourier patterns. The filtered images were aligned by rigid registration using SmartAlign (HREM Research Inc.),^30^ followed by moving average filtering along the time axis with a window size of two.

### Image Simulation

Crystallographic data of the 1T″ structure from the literature^23^ were used to construct structural models for the bulk and a domain structure. The sizes for the former and the latter were 5.3 × 4.7 × 15.0 and 11.5 × 10.0 × 15.0 nm, respectively. ADF-STEM image simulations were performed using the PRISM algorithm.^31,32^ For the bulk, the interpolation factor was 1, and for the domain structure, it was 3. In both cases, the slice thickness was 0.2 nm and Gaussian blurring with a sigma value of 0.09 nm was applied. The center regions with dimensions of 4.9 × 4.4 and 11.1 × 9.6 nm of the simulated images of the bulk and domain structure models were cropped, respectively.

## Supplementary Material















## Notes

EELS analysis of air-exposed Li, additional note about experimental setup, ADF-STEM images demonstrating necessity of electron irradiation for MoS_2_ lithiation, conceptual schematics of energy levels of possible lithiation reactions, temporal evolution of lithiated areas, examination of C and O migration, processed high-magnification ADF-STEM images of MoS_2_ lithiation, simulated ADF-STEM image of lithiated MoS_2_, note about model thickness for image simulation, and evaluation of lattice expansion (PDF)
ADF-STEM movie demonstrating necessity of electron irradiation for MoS_2_ lithiation (MP4)
Low-magnification ADF-STEM movie of MoS_2_ lithiation under alternating electron dose rates (MP4)
Low-magnification ADF-STEM movie of MoS_2_ lithiation under a constant electron dose rate (MP4)
Movie showing alternately acquired low-magnification BF- and high-magnification ADF-STEM images during MoS_2_ lithiation (MP4)
Movie showing processed high-magnification ADF-STEM images of MoS_2_ lithiation (MP4)
Movie showing the domain structure model used for image simulation (MP4)
