# Supplementary material for: Atomic-Scale In Situ Scanning Transmission Electron Microscopy of MoS2 during Lithiation
Source: ACS Nano. 2025 Jul 21;19(30):27332–7. doi: 10.1021/acsnano.5c05218 (PMC12333416; doi:10.1021/acsnano.5c05218)
Supplement: Supplementary file 1 [file nn5c05218_si_001.pdf]

Supporting Information

Atomic-Scale In Situ Scanning Transmission Electron Microscopy of MoS_2 during Lithiation

Kei Nakayama and Shunsuke Kobayashi

Nanostructures Research Laboratory, Japan Fine Ceramics Center, Nagoya, Aichi 456-8587, Japan

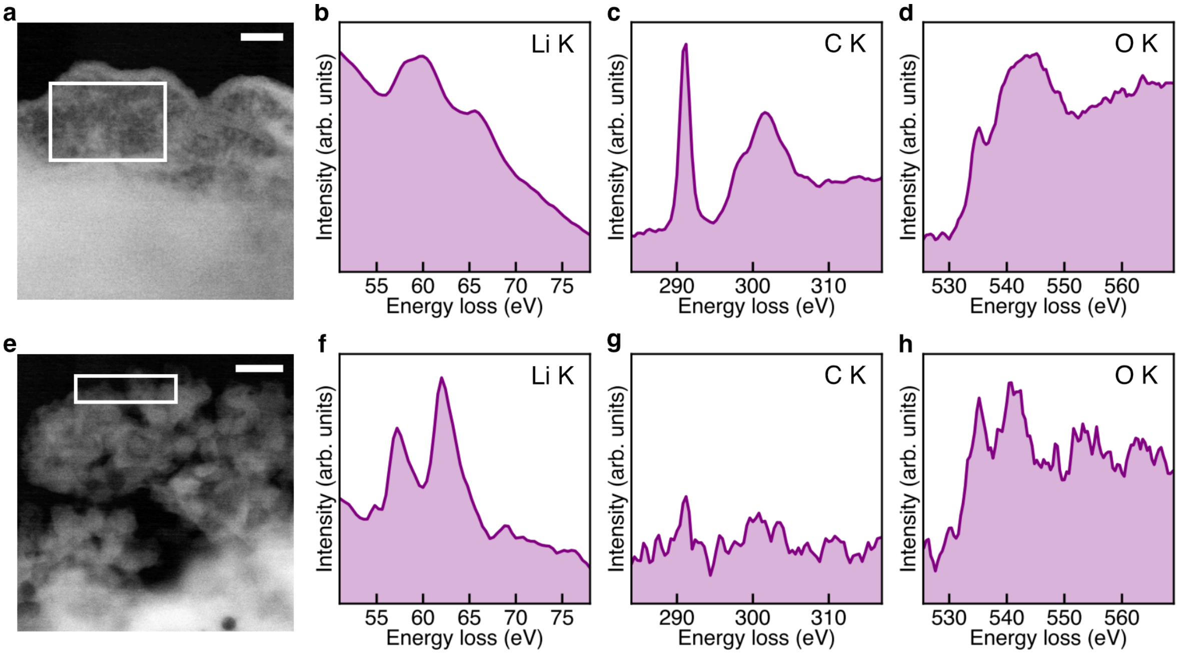

Figure S1. EELS analysis of Li exposed to air for approximately 30 minutes. (a) ADF-STEM image of air-exposed Li on the W probe. (b–d) EEL spectra of the rectangular region in (a). The Li K-, C K-, and O K-edges are visible, whose shapes are similar to those of Li_2CO_3 in the literature.[1-3] (e) ADF-STEM image of air-exposed Li on the W probe. (f–h) EEL spectra of the rectangular region in (e). The Li K- and O K-edges are dominant, whose shapes are similar to those of Li_2O in the literature.[3,4] Li_2CO_3 and Li_2O were presumably generated by the reactions of Li with CO_2 and O_2 in air, respectively.[5,6] Scale bars, 100 nm (a) and 50 nm (e).

Note S1. Additional information on the experimental setup. The lithiation of MoS_2 likely occurred concurrently with the decomposition of the air-exposed Li induced by electron irradiation. Possible reactions are MoS_2 + 1/2 Li_2CO_3 → $LiMoS_2$ + 1/4 O_2 + 1/2 CO_2 and MoS_2 + 1/2 Li_2O → $LiMoS_2$ + 1/4 O_2. This proposal is based on the following considerations:

(1) The dominant component of the air-exposed Li was Li_2CO_3 (Figure S1a–d) with a small amount of Li_2O (Figure S1e–h). The observed frequency of Li_2O was less than one-tenth that of Li_2CO_3.Considering the high reactivity of Li metal, the thickness of the air-exposed Li on the W probe (a few micrometers at most), and the exposure time to air (approximately 30 min), it is reasonable to assume that almost no Li metal remains in the air-exposed Li. Therefore, Li_2CO_3 and Li_2O, rather than Li metal, are suggested as the Li sources for the lithiation of MoS_2.

(2) As shown in Figure S2 and Movie S1, electron irradiation was necessary for MoS_2 lithiation. In terms of the Gibbs free energy, the two possible reactions above are consistent with the necessity of electron irradiation, as schematically illustrated in Figure S3.

Unfortunately, we were unable to identify any significant difference between Li_2CO_3 and Li_2O in terms of their effects on the lithiation behavior of MoS_2. This was partly due to the difficulty in accurately determining the compounds present in the air-exposed lithium specifically at the MoS_2 interface, and partly due to the inherently small amount of Li_2O.

The lithiated area increased linearly with time, suggesting that lithiation was induced at a constant rate by electron irradiation applied at regular intervals in STEM (Figure S4).

The lithiation process may be saturated with respect to the electron dose rate (Figure S5).

No significant migration of C and O into MoS_2 was observed (Figure S6).

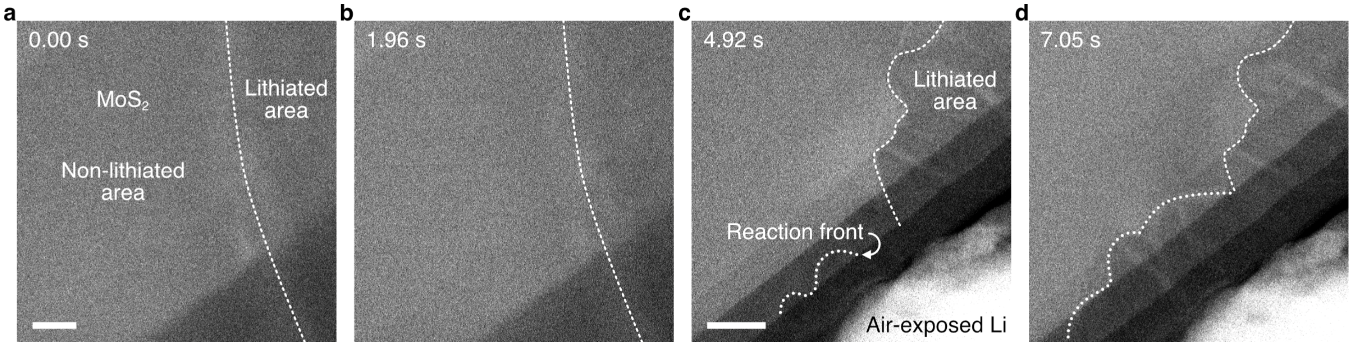

Figure S2. Snapshots from Movie S1, acquired using ADF-STEM. The area on the right side of the 0.00 s image in (a) was lithiated before the series of images was acquired. Up to 1.96 s, the electron beam is irradiated only onto the interior of the MoS_2 specimen, with no significant changes observed. Between 1.96 and 4.74 s, the magnification is lowered, and the electron beam irradiates the area around the MoS_2/air-exposed Li interface. At 4.92 s, the initiation of the lithiation process becomes visible, with the lithiated region expanding into the MoS_2 specimen until 7.05 s. Scale bars, 5 nm (a) and 50 nm (c).

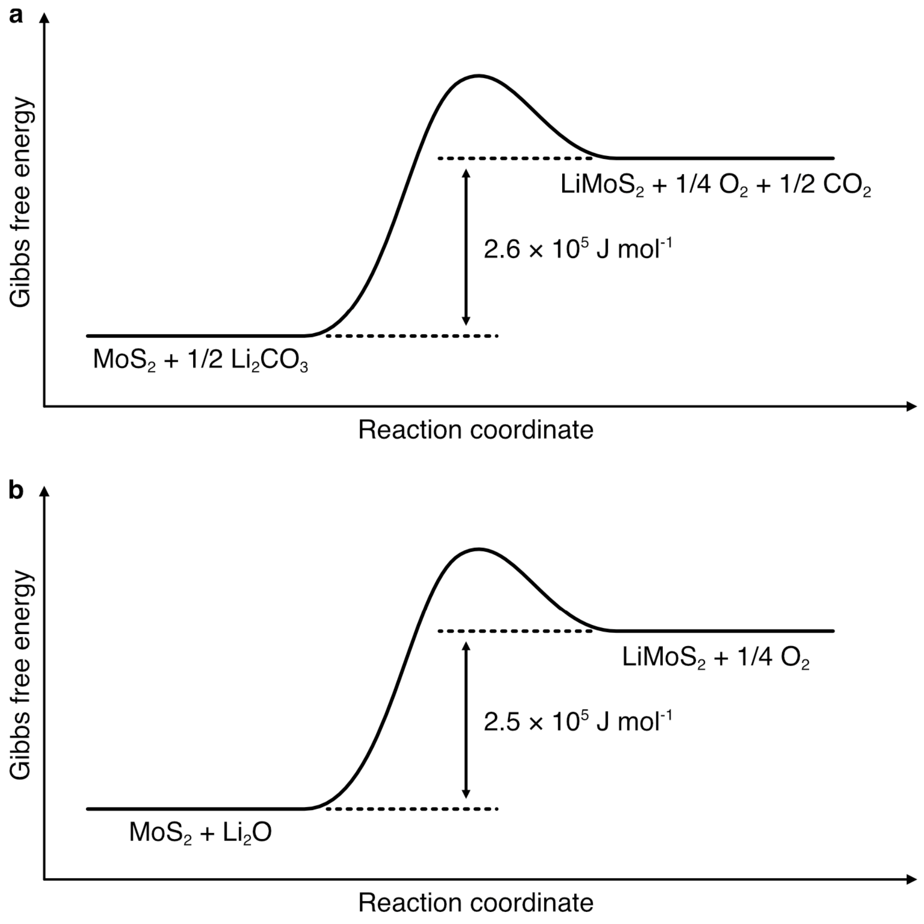

Figure S3. Conceptual schematics of the Gibbs free energy for the two proposed lithiation reactions in our experimental setup. (a) $MoS_2 + 1/2\ Li_2CO_3 \rightarrow LiMoS_2 + 1/4\ O_2 + 1/2\ CO_2$ and (b) $MoS_2 + Li_2O \rightarrow LiMoS_2 + 1/4\ O_2$. Based on the calculated internal energy of formation for the solid phases and thermodynamic data for the gas phases,[7,8] the right sides are estimated to be higher than the left sides on the order of 10^5 J mol^{-1}, indicating that the proposed reactions do not occur spontaneously. However, 80-kV-accelerated electrons (7.7×10^9 J mol^{-1}) may induce these reactions by transferring a part of their energy. Note that the proposed reactions produce O_2 and CO_2 gases, which diffuse into the vacuum of the microscope (< 2×10^{-5} Pa). Therefore, the reverse reactions presumably hardly take place although the left sides are more energetically favorable than the right sides.

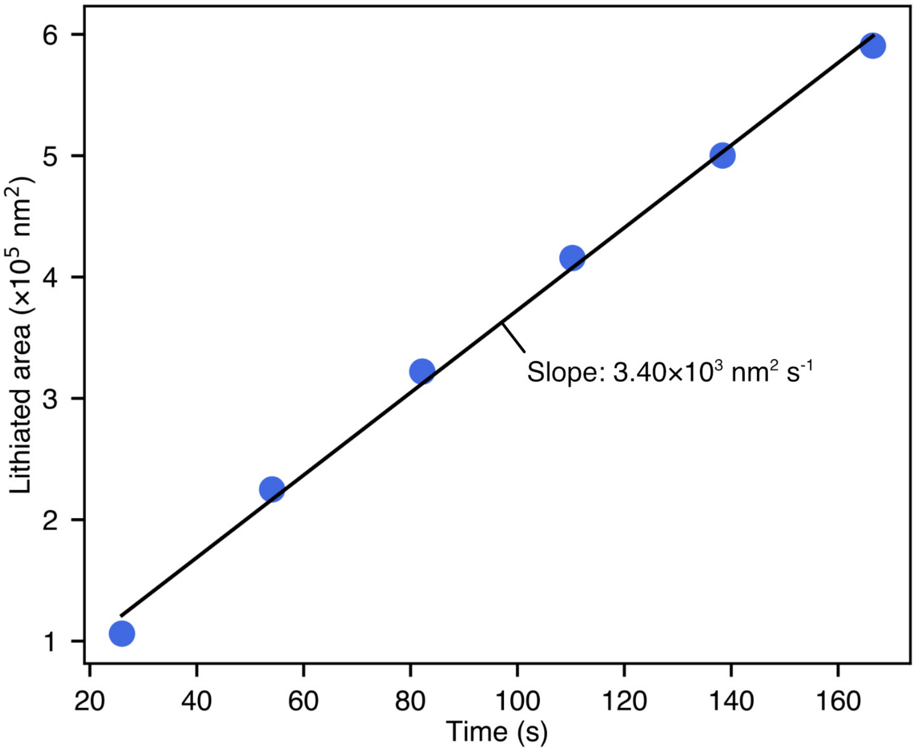

Figure S4. Temporal evolution of the lithiated area shown in Figure 1 of the main text and in Movie S3. The points indicate the measured areas, and the solid line shows the result of the fitting.

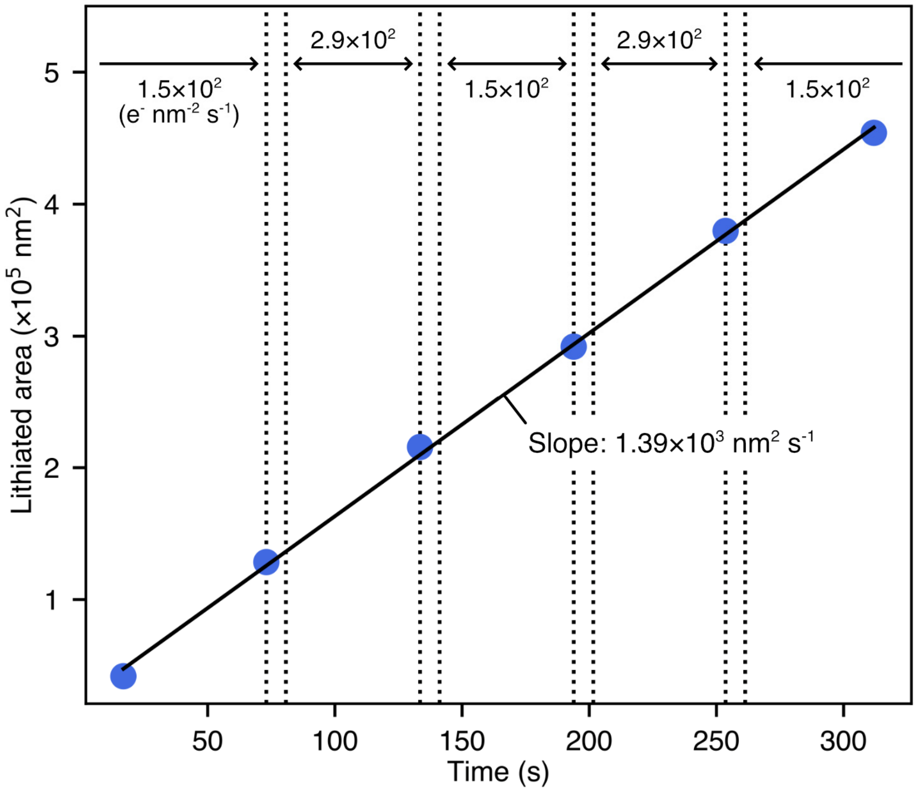

Figure S5. Temporal evolution of the lithiated area under alternating electron dose rates. The corresponding ADF-STEM images are shown in Movie S2. The dose rate was varied between 1.5×10^2 and 2.9×10^2 e^- nm^{-2} s^{-1}, values comparable to the constant dose rate used in Figure S4 (1.8×10^2 e^- nm^{-2} s^{-1}). As shown in the graph, no significant effect of the dose rate variation on the lithiation rate was observed, suggesting that the lithiation process was saturated with respect to the electron dose rate. Notably, the lithiation rate (1.39×10^3 nm^2 s^{-1}) is substantially lower than that observed in Figure S4 (3.40×10^3 nm^2 s^{-1}), indicating that, with the electron dose rate already ruled out, a factor other than Li diffusivity within Li_xMoS_2 may be rate-limiting. A plausible limiting factor is the contact area between MoS_2 and air-exposed Li, although this parameter is difficult to estimate accurately from two-dimensional images.

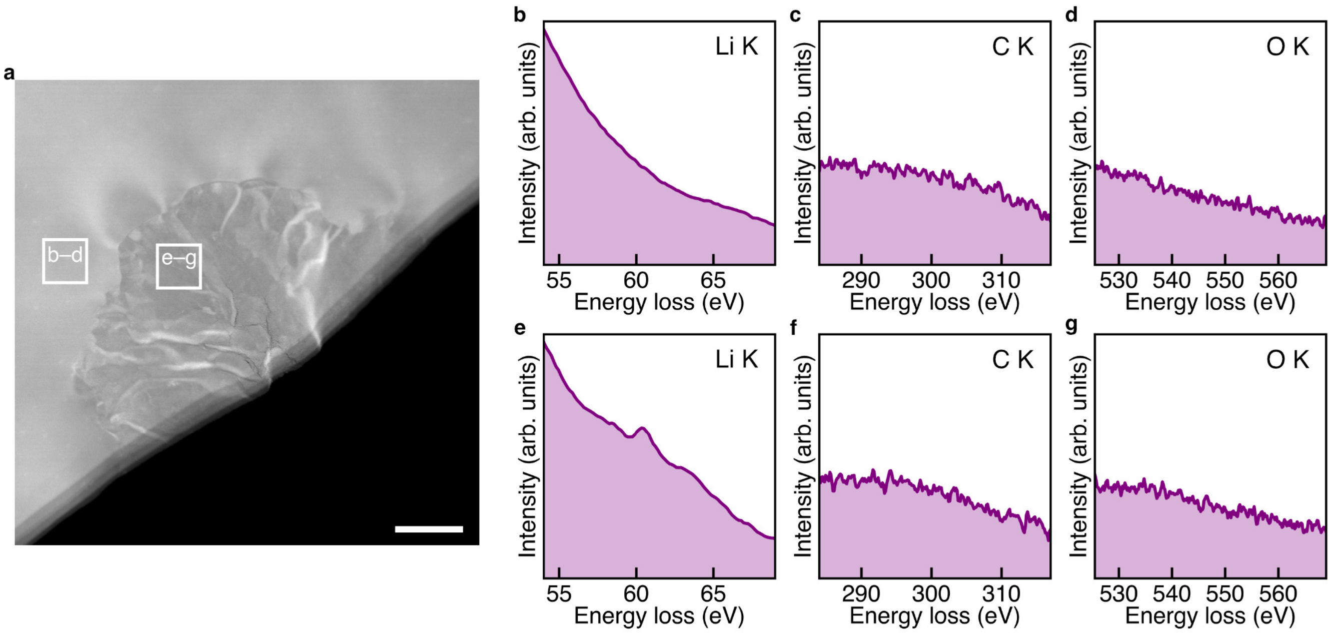

Figure S6. (a) ADF-STEM image of lithiated MoS_2, acquired from the same region shown in Movie S2. (b–g) EEL spectra obtained from the square regions in (a). No significant amount of C and O was detected in the lithiated area. Scale bar, 200 nm (a).

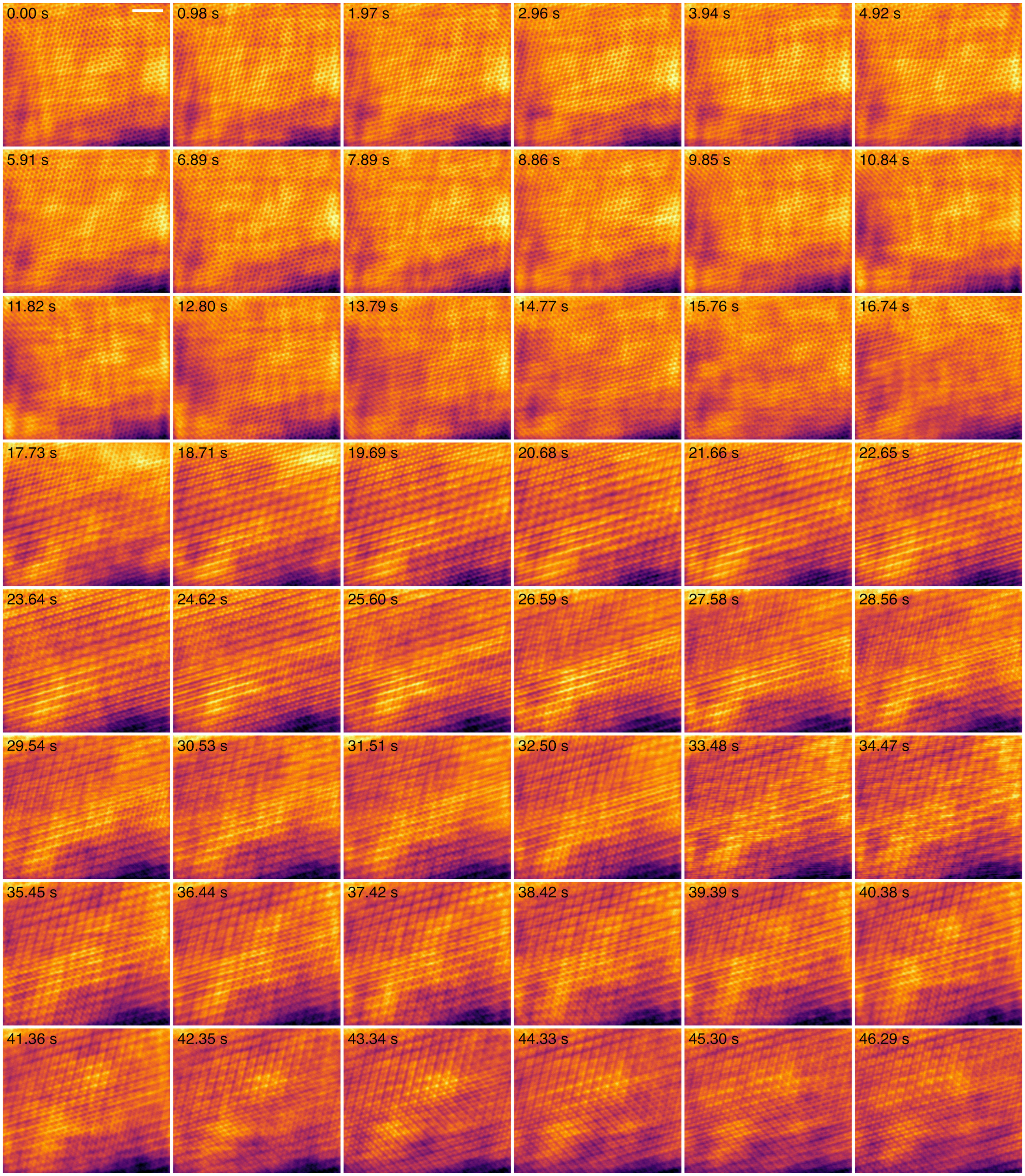

Figure S7. In situ ADF-STEM images of MoS_2 lithiation, processed with threshold filtering in reciprocal space, image alignment, and moving average filtering. Scale bar, 2 nm.

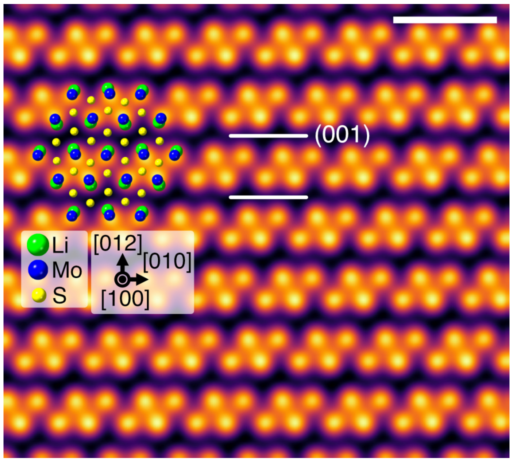

Figure S8. Simulated ADF-STEM image of the 1T″ structure.[9] Scale bar, 1 nm.

Note S2. Reason behind the model thickness for Figure 3e,f in the main text. The relative thickness of the MoS_2 specimen, t/λ, where t is the absolute thickness and λ is the inelastic mean free path of the incident electrons, was estimated using the log-ratio method in EELS.[10] For the particularly thin region (approximately 50 nm from the specimen edge) used for high-magnification ADF-STEM observation, the relative thickness was typically in the range of 0.2 to 0.25. Using a rough estimation of $\lambda \approx 0.8 \times E_0$,[10] where λ is in nm and E_0 is the incident electron energy in keV (i.e., 80 keV in our case), the corresponding absolute thickness was estimated to be around 15 nm.

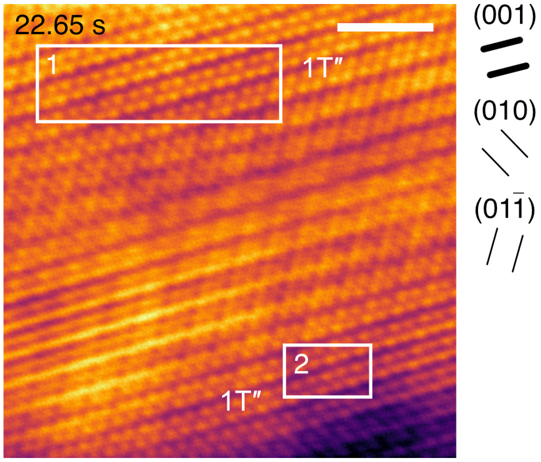

Figure S9. Central region of the 22.65 s image (shown in both Movie S5 and Figure S7), showing two 1T″ regions in the upper left and lower right, separated by an inclined, out-of-phase boundary. Both regions have the same crystallographic orientation, and the directions of the (001), (010), and (01$\bar{1}$) planes are indicated, respectively, by the pairs of lines on the right side of the image. Using geometric phase analysis[11,12] with a reference image of non-lithiated 2H structure (the central region of Figure 2b in the main text), the average lattice expansions normal to the (001), (010), and (01$\bar{1}$) planes in rectangular region 1 were estimated to be approximately 4.3%, 2.2%, and 1.1%, respectively. Those in rectangular region 2 were estimated to be approximately 2.6%, 1.1%, and 1.4%, respectively. A possible reason why these values are lower than those anticipated from the reported crystallographic data[9,13] (7.5%, 5.6%, and 5.4%) is the constraint from the parent 2H phase. Nevertheless, the results demonstrate that lithiation tends to induce greater lattice expansion along the direction normal to the (001) plane in the 1T″ structure. Scale bar, 2 nm.

References

(1) Lin, F.; Markus, I. M.; Doeff, M. M.; Xin, H. L., Chemical and Structural Stability of Lithium-Ion Battery Electrode Materials under Electron Beam. *Sci. Rep.* **2014**, *4*, 5694.

(2) Wang, F.; Graetz, J.; Moreno, M. S.; Ma, C.; Wu, L.; Volkov, V.; Zhu, Y., Chemical Distribution and Bonding of Lithium in Intercalated Graphite: Identification with Optimized Electron Energy Loss Spectroscopy. *ACS Nano* **2011**, *5*, 1190–1197.

(3) Fister, T. T.; Schmidt, M.; Fenter, P.; Johnson, C. S.; Slater, M. D.; Chan, M. K. Y.; Shirley, E. L., Electronic Structure of Lithium Battery Interphase Compounds: Comparison between Inelastic X-ray Scattering Measurements and Theory. *J. Chem. Phys.* **2011**, *135*, 224513.

(4) Zachman, M. J.; Tu, Z.; Choudhury, S.; Archer, L. A.; Kourkoutis, L. F., Cryo-STEM Mapping of Solid–Liquid Interfaces and Dendrites in Lithium-Metal Batteries. *Nature* **2018**, *560*, 345–349.

(5) Zheng, S.; Geng, H.; Eliseeva, S. N.; Wang, B., Air-Exposed Lithium Metal as a Highly Stable Anode for Low-Temperature Energy Storage Applications. *Energy Mater.* **2022**, *2*, 200042.

(6) Liu, Y.; Wang, L.; Cao, L.; Shang, C.; Wang, Z.; Wang, H.; He, L.; Yang, J.; Cheng, H.; Li, J.; Lu, Z., Understanding and Suppressing Side Reactions in Li–Air Batteries. *Mater. Chem. Front.* **2017**, *1*, 2495–2510.

(7) Jain, A.; Ong, S. P.; Hautier, G.; Chen, W.; Richards, W. D.; Dacek, S.; Cholia, S.; Gunter, D.; Skinner, D.; Ceder, G.; Persson, K. A., Commentary: The Materials Project: A Materials Genome Approach to Accelerating Materials Innovation. *APL Mater.* **2013**, *1*, 011002.

(8) P.J. Linstrom and W.G. Mallard, Eds., NIST Chemistry WebBook, DOI: 10.18434/T4D303.

(9) Schwarzmüller, S.; Wurst, K.; Heymann, G.; Huppertz, H., Pressure-Assisted Synthesis of Highly Crystalline 1*T*″-Li_xMoS_2. *Chem. Eur. J.* **2024,** *30*, No. e202302565.

(10) Egerton, R. F., *Electron Energy-Loss Spectroscopy in the Electron Microscope*; Springer, 2011.

(11) Hÿtch, M. J.; Snoeck, E.; Kilaas, R., Quantitative Measurement of Displacement and Strain Fields from HREM Micrographs. *Ultramicroscopy* **1998,** *74*, 131–146.

(12) de Jong, T. A.; van der Molen, S. J., pyGPA, DOI: 10.5281/zenodo.5589555.

(13) McMurdie, H. F.; Morris, M. C.; Evans, E. H.; Paretzkin, B.; Wong-Ng, W.; Hubbard, C. R., Standard X-Ray Diffraction Powder Patterns from The JCPDS Research Associateship. *Powder Diffr.* **1986,** *1*, 265–275.
